# A mathematical model of iron import and trafficking in wild-type and Mrs3/4ΔΔ yeast cells

**DOI:** 10.1186/s12918-019-0702-2

**Published:** 2019-02-21

**Authors:** Joshua D. Wofford, Paul A. Lindahl

**Affiliations:** 1 Texas A&M University, Department of Chemistry, College Station, TX 77843-3255 USA; 2 Texas A&M University, Department of Biochemistry & Biophysics, College Station, 77843-3255 USA

**Keywords:** Ordinary differential equations, Nanoparticles, Iron-sulfur clusters, Mitochondria, Vacuoles, Cytosol, Kinetics, Oxygen

## Abstract

**Background:**

Iron plays crucial roles in the metabolism of eukaryotic cells. Much iron is trafficked into mitochondria where it is used for iron-sulfur cluster assembly and heme biosynthesis. A yeast strain in which Mrs3/4, the high-affinity iron importers on the mitochondrial inner membrane, are deleted exhibits a slow-growth phenotype when grown under iron-deficient conditions. However, these cells grow at WT rates under iron-sufficient conditions. The object of this study was to develop a mathematical model that could explain this recovery on the molecular level.

**Results:**

A multi-tiered strategy was used to solve an ordinary-differential-equations-based mathematical model of iron import, trafficking, and regulation in growing *Saccharomyces cerevisiae* cells. At the simplest level of modeling, all iron in the cell was presumed to be a single species and the cell was considered to be a single homogeneous volume. Optimized parameters associated with the rate of iron import and the rate of dilution due to cell growth were determined. At the next level of complexity, the cell was divided into three regions, including cytosol, mitochondria, and vacuoles, each of which was presumed to contain a single form of iron. Optimized parameters associated with import into these regions were determined. At the final level of complexity, nine components were assumed within the same three cellular regions. Parameters obtained at simpler levels of complexity were used to help solve the more complex versions of the model; this was advantageous because the data used for solving the simpler model variants were more reliable and complete relative to those required for the more complex variants. The optimized full-complexity model simulated the observed phenotype of WT and Mrs3/4ΔΔ cells with acceptable fidelity, and the model exhibited some predictive power.

**Conclusions:**

The developed model highlights the importance of an Fe^II^ mitochondrial pool and the necessary exclusion of O_2_ in the mitochondrial matrix for eukaryotic iron-sulfur cluster metabolism. Similar multi-tiered strategies could be used for any micronutrient in which concentrations and metabolic forms have been determined in different organelles within a growing eukaryotic cell.

**Electronic supplementary material:**

The online version of this article (10.1186/s12918-019-0702-2) contains supplementary material, which is available to authorized users.

## Background

The complexity of biochemical processes in growing eukaryotic cells is enormous, often rendering the corresponding genetic phenotypes difficult to understand at the chemical level. One means of analyzing such systems is to develop ordinary-differential-equation (ODE^1^)-based kinetic models [1–3]. In principle, such models can reveal on a quantitative basis whether observed phenotypic behavior could emerge from a proposed system of reacting chemical players using a particular set of kinetic and thermodynamic parameters. This is a huge advantage relative to the common practice of describing complex biochemical processes as a cartoon or scheme. Another advantage of math-based kinetic models is that all assumptions are explicit and available for public inspection whereas cartoons and schemes generally include hidden assumptions. The major *disadvantage* of such kinetic models is that a complete and accurate dataset, including rate-law expressions, rate-constants, and reactant concentrations, are required to solve them and to endow them with predictive power. Rarely is all such information available, and available information is often less quantitative than desired.

A common approach to circumventing this problem is to employ *simple* models (in terms of numbers of components and reactions) that nevertheless remain capable of generating observed cellular behavior and of explaining genetic phenotypes. Designing such models involves deciding which species and reactions to include, which to leave out, and which to combine into groups. Such decisions often boil-down to whether including an additional component or reaction is “worth” (in terms of generating the desired behavior) an additional adjustable parameter. Simple models with few adjustable parameters simplify reality but they can also provide fundamental insights into reality - by penetrating through the entangled and bewildering complexity of a highly complex system.

Iron is critical for all eukaryotic cells [4, 5]. It is present in many forms including heme centers, iron-sulfur clusters (ISCs), nonheme mononuclear species, and iron-oxo dimeric centers. Such centers are commonly found in the active-sites of metalloenzymes. Iron plays a major role in energy metabolism; e.g. there are iron-rich respiratory complexes located on the inner membrane of mitochondria. Mitochondria are the primary site in the cell where ISCs are assembled, and the only site where iron is installed into porphyrins during heme biosynthesis. For these reasons, mitochondria are a major ‘hub’ for iron trafficking.

The cytosol also plays an important role in iron trafficking, in that nutrient iron enters this region prior to being distributed to the organelles. Most of the iron that enters the cytosol is probably in the Fe^II^ state, but neither the oxidation state nor the concentration of cytosolic Fe has been established [6]. The vacuoles are another trafficking ‘hub’ in yeast, as much of the iron imported into these cells (when grown on iron-sufficient media) is stored in these acidic organelles [7, 8]. Vacuolar iron is predominately found as a mononuclear nonheme high spin (NHHS) Fe^III^ species, probably coordinated to polyphosphate ions [9].

Iron is tightly regulated in cells, and some insightful mathematical models involving iron metabolism, trafficking and regulation have been developed. Twenty years ago, Omholt et al. designed and analyzed a model of the IRP/IRE iron regulatory system in mammalian cells [10]. More recently, Mobilia et al. developed a similar model that assumed scarce or unavailable data; they also developed new methods to represent data by constrained inequalities [11, 12]. Chifman and coworkers developed an ODE-based model for iron dysregulation in cancer cells in which the roles of the IRP-based regulation, the iron storage protein ferritin, the iron export protein ferroportin, the labile iron pool, reactive oxygen species, and the cancer-associated Ras protein were emphasized [13], as well as a logical-rule-based mathematical model of iron homeostasis in healthy mammalian cells [14]. Mitchell and Mendes used ODE’s to model iron metabolism and regulation in a liver cell and its interaction with blood plasma [15]. They emphasized the role of iron-regulating hormone hepcidin and the regulatory and storage systems mentioned above, and they simulated the effects of iron-overload disease. Their model was complex - involving 66 adjustable parameters many of which were not experimentally determined. None of the above models included iron-sulfur cluster (ISC) synthesis, the role of mitochondria (or other organelles), and none modeled growing cells. In terms of biological emphasis, the model of Achcar et al. [16] is most relevant to the current study. They developed a model of iron metabolism and oxidative stress in yeast cells using a Boolean approach using weighted reactions. Their model included ISC assembly, as well as organelles such as mitochondria, vacuoles, cytosol, and nucleus. However, their model was exceedingly complicated (642 components and 1007 reactions) and was not ODE based [16]. They modeled the development of Fe^III^ (phosphate) oxyhydroxide nanoparticles in mitochondria of mutant cells lacking ISC assembly proteins (e.g. Yfh1, the yeast frataxin homolog), similar to the emphasis of our previous model [17]. They included a reaction in which an unidentified species X converted nanoparticles into free iron, and hypothesized that X might be glutathione. In contrast, our model emphasized the role of oxygen in controlling nanoparticle formation.

The iron content of yeast cells and the major organelles involved in iron trafficking have been analyzed using Mössbauer (MB) spectroscopy, the most powerful spectroscopic tool for interrogating the iron content of biological samples [18]. If the absolute iron concentration of ^57^Fe-enriched cells and organelles are known, the absolute concentrations of major groups of iron-containing species in such cells can be calculated using percentages obtained by MB. Such data is used here to develop an advanced mathematical model of iron import and trafficking in eukaryotic cells.

In WT cells, much iron enters mitochondria through Mrs3 and Mrs4, paralogous inner-membrane proteins [19, 20]. These “high affinity” iron-importers contain a small tunnel that allows a low-molecular-mass cytosolic iron species to enter the matrix. We have recently discovered a low-molecular-mass species in mitochondria, designated Fe_580_, which might serve as feedstock for ISC assembly [21]. Iron can enter mitochondria through alternative pathways, including one that involves Rim2 [22].

Iron import in yeast is regulated according to the ISC activity occurring in mitochondria [23]. When this activity is attenuated, for example by mutations in the ISC assembly machinery, the rate of nutrient iron imported increases. In yeast, iron regulation involves the *Iron Regulon*, a group of 20–30 genes whose expression is controlled by transcription factors Aft1/2 [24, 25]. This includes the Fet3/Ftr1 complex on the plasma membrane through which much iron enters the cell.

Yfh1 helps catalyze ISC assembly in mitochondria [26]. This and other ISC mutant cells accumulate large quantities of iron in the form of Fe^III^ nanoparticles [27, 28]. These cells import excessive iron because the iron regulon is activated in response to insufficient mitochondrial ISCs. Excess iron (in the form of nanoparticles) accumulates in mitochondria because the rate of iron import into the organelle increases due to activation of the iron regulon. The *net* rate of iron import into vacuoles is reduced such that these organelles contain little iron in ISC mutants. Actually, the iron *export* rate is probably *increased* in these mutants. The vacuolar membrane contains an iron-export complex (Fet5/Fth1) that is homologous to the Fet3/Ftr1 iron import complex on the plasma membrane; both are controlled by the iron regulon [29].

**Fig. 1:**
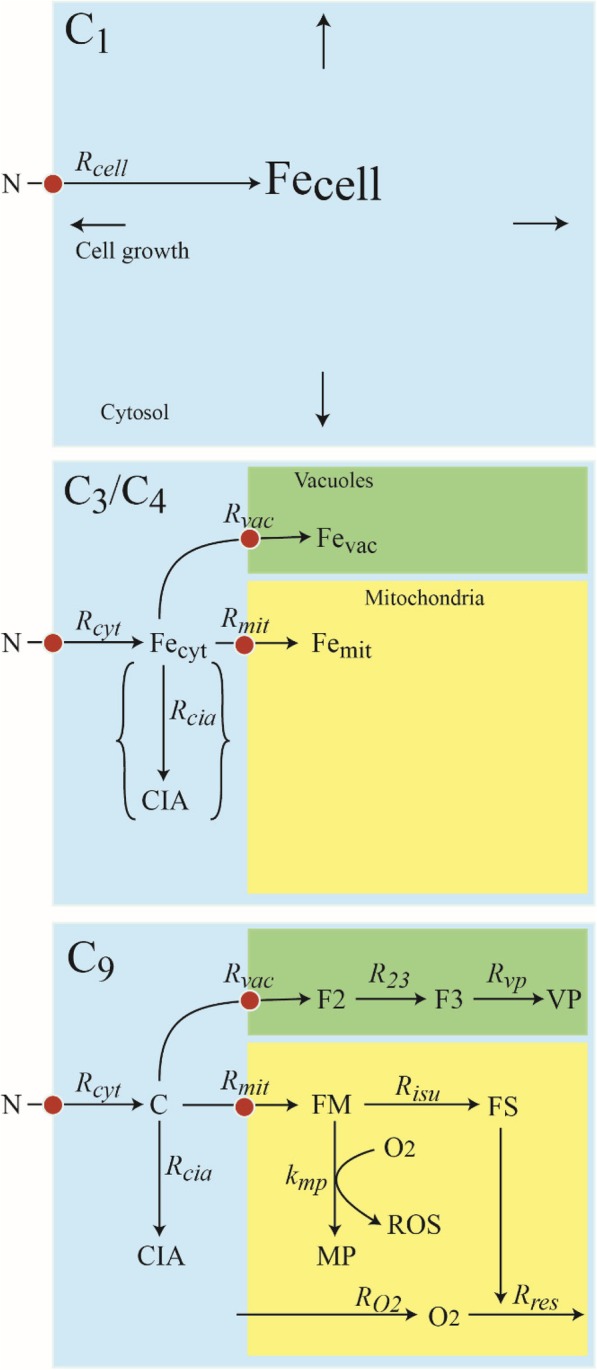
Strategy for optimizing a model of nutrient iron import, trafficking and regulation in growing eukaryotic cells. Top panel: C_1_ model in which all iron in the cell is treated as a single species and the cell is considered to be homogeneous. Middle panel: C_3_ model in which the cell is divided into three regions and each region is assumed to contain a single type of iron species. C_4_ model includes the reaction forming *CIA*. Bottom panel: C_9_ model in which the cell remains divided into 3 regions but the number of iron-containing species is expanded to 8

We have developed a simple model (Fig. 1, bottom panel) to illustrate the changes in iron import and trafficking that occur in ISC mutants relative to in WT cells [17]. The core assumption of the model is that the matrix of healthy WT mitochondria is *micro-aerobic*, containing less O_2_ than in an aerobic state due to the ability of the respiratory complexes on the inner membrane to quickly reduce much of the O_2_ that would otherwise diffuse into the matrix. Although dissolved [O_2_] concentrations in the mitochondrial matrix have not been measured directly, three lines of evidence indicate that this space is micro-aerobic. Firstly, in vitro ISC assembly assays must be performed anaerobically because some of the proteins involved are O_2_-sensitive [30]. Secondly, numerous other enzymes in the matrix, including aconitase, biotin synthase, and lipoic acid synthase are O_2_-sensitive [31, 32]. Thirdly, the nitrogenase iron protein which is exquisitely O_2_-labile remains active when installed in the mitochondrial matrix [33]. According to our model, in ISC mutant mitochondria, the lack of ISCs and heme centers cause a deficiency of respiratory complexes; this condition allows O_2_ to diffuse into the matrix and react with a pool of Fe^II^, forming nanoparticles.

Relative to WT cells, Mrs3/4ΔΔ cells (to be called **ΔΔ** hereafter) grow slowly under iron-deficient conditions but at WT rates in iron-sufficient media [5, 19, 20]. The iron concentration of ΔΔ cells is higher than in comparable WT cells, indicating that the iron regulon is activated. We recently found that mitochondria from iron-deficient ΔΔ cells are dominated by nanoparticles whereas the iron content of mitochondria from iron-sufficient ΔΔ cells are similar to WT mitochondria – i.e. dominated by the ISC and heme centers that are found in respiratory complexes. WT mitochondria also contain a substantial amount of a NHHS Fe^II^ that probably arise from Fe_580_ [34]. Fe_580_ is present in mitochondria from iron-replete ΔΔ and both iron-deficient and iron-replete WT cells. However, our previous model [17] was unable to reproduce the ΔΔ phenotype.

In this paper, we present an improved ODE-based model of iron trafficking and regulation in yeast, and use a multi-tiered strategy to solve it at an expanding steady-state. This model was able to explain both the ΔΔ *and* ΔYfh1 phenotypes while requiring fewer adjustable parameters relative to our previous model.

## Methods and results

As is typical of modeling biochemical processes within cells, the challenge was to generate a useful and insightful model despite sparse and imperfect data [11, 12]. Our strategy for doing this was to optimize the model at different levels of complexity. Model variants ranged from one that consisted of a single iron species and no cellular compartments to one that involved nine species in three cellular compartments. The parameters used to optimize the simpler variants were transferrable to the more complex models. This was an important insight because the data needed to solve simpler systems tend to be more reliable and complete relative to those required to solve more complex variants. A similar strategy could be applied for models involving the trafficking of other micronutrients. The only requirements are that the concentrations and metabolic forms of the micronutrient in the cell and in major organelles be known (at some reasonable level of accuracy) for different growth conditions and/or genetic strains.

The complete chemical model is shown in Fig. 1, bottom panel. We initially solved it (to be referred to as ***C***_***9***_, the “nine-component” model, including components *C*, *CIA*, *F2*, *F3*, *VP*, *FM*, *FS*, *MP*, and *O*_*2*_) at three simpler levels of complexity called ***C***_***1***_ (the “one-component” model, with component *Fe*_*cell*_), ***C***_***3***_ (the “three-component” model, including *Fe*_*cyt*_, *Fe*_*mi*t_, and *Fe*_*vac*_), and ***C***_***4***_ (the “four-component” model, including *C*, *CIA*, *Fe*_*vac*_, and *Fe*_*mit*_). These model variants are illustrated in Fig. 1, top and middle panels. We solved C_9_ in this way because the data required to solve the simpler versions were more reliable and complete than those required to solve the C_9_ variant. Importantly, *the parameters that were optimized using the simpler versions could be transferred to the more complex variants*. This minimized the number of adjustable parameters that had to be assigned using less reliable or incomplete data. As far as we are aware, this multi-tiered modeling strategy has not been employed previously within the context of ODE-based models involving the trafficking of iron or any micronutrient within a growing eukaryotic cell. Code for all model variants was written using *Mathematica 10* software (wolfram.com). Initial concentrations for each iron component was 10 μM, and initial [O_2_] was 0 μM. ODEs were solved to steady-state using the NDSolve routine.

### Development of the C_1_ model

Consider a population of cells growing exponentially on a nutrient form of iron called *N* which enters the cell through a transporter on the plasma membrane (Fig. 1, top panel, red circle). In the experimental results used here in fitting [34], N consisted of 0, 1, 10 and 40 μM ferric citrate plus 1 μM endogenous iron as found in minimal medium. Let *V*_*cell*_ represent the collective cell volume (within a culture) at time *t*. When cells are growing exponentially, *V*_*cell*_ will increase according to the relationship.

1 $$ \frac{dV_{cell}}{dt}={\alpha}_{cell}\cdot {V}_{cell} $$

where *α*_*cell*_ is the growth rate. During exponential growth *α*_*cell*_ is constant in time. The optical density at 600 nm of an exponentially growing culture is proportional to *V*_*cell*_ such that the slope of the {ln(OD_600_) vs. time} plot affords *α*_*cell*_. This parameter has been determined for WT and ΔΔ cells grown in medium containing [N] = 1, 2, 11, and 41 μM ([34] and Table 1). The 8 “data-based” determinations of *α*_*cell*_ will be called *α*_*cell* − *dat*_.

**Table 1:** Growth rates, iron concentrations, and import rates in ΔΔ and WT cells grown under different nutrient conditions. [N] refers to the μM concentration of iron in the respiring medium, as described [34]. The untreated medium was assumed to contain 1 μM of endogenous iron. For each entry, the top number is datum or data-based estimates (*R*_*…-dat*_) and the bottom number is the corresponding simulated value (*R*_*…-sim*_). Concentrations are in units of μM, rates are in units of μM/hr., and α_cell_ is in units of hr.^− 1^. Data for α_cell_ and [Fe_cell_] have been published [34] whereas [Fe_cyt_], [Fe_mit_], and [Fe_vac_] were estimated as described in the text

[N]	α_cell_	[Fe_cell_]	*R*_*cell*_	*f*_*cyt*_⋅[Fe_cyt_]	*f*_*mit*_ ⋅[Fe_mit_]	*f*_*vac*_ ⋅[Fe_vac_]	*R*_*cyt*_ *- R*_*mit*_ *- R*_*vac*_	*R*_*mit*_	*R*_*vac*_
WT									
1	0.18	120	22	79	41	0	18	9.2	0
	0.18	60	10	22	22	16	5.0	4.9	3.7
2	0.18	200	37	75	43	82	17	9.9	19
	0.19	190	35	76	35	81	18	8.4	19
11	0.20	480	97	200	56	220	52	14	56
	0.20	810	160	180	66	560	45	17	140
41	0.20	880	180	310	69	500	77	18	130
	0.20	900	180	180	69	640	46	18	160
ΔΔ									
1	0.06	360	22	110	69	180	8.1	5.2	14
	0.05	540	27	240	74	230	13	3.9	12
2	0.068	680	46	130	69	480	11	5.9	41
	0.069	1100	90	380	77	660	33	6.6	58
11	0.15	2200	320	280	72	1800	52	13	340
	0.15	2300	360	280	71	1900	53	13	360
41	0.20	3900	800	230	74	3600	59	19	920
	0.19	2000	390	230	61	1800	54	14	410

For simulations, a continuous α function between *N* = 1–41 μM was required. Plots of *α*_*cell*_ vs. [N] exhibited saturation behavior, suggesting the Michaelis-Menten type function

2 $$ {\alpha}_{cell- sim}=\frac{\alpha_{\mathrm{max}}\left[N\right]}{K_{\alpha }+\left[N\right]}. $$

The desired continuous α function was obtained by fitting (2) against the *α*_*cell* − *dat*_values using the error function.

3 $$ ERR=\frac{1}{4}\sum \limits_{N=1,2,11,41}\frac{2\cdot \left|{\alpha}_{cell- sim,N}-{\alpha}_{cell- dat,N}\right|}{\alpha_{cell- sim,N}+{\alpha}_{cell- dat,N}}. $$

**Fig. 2:**
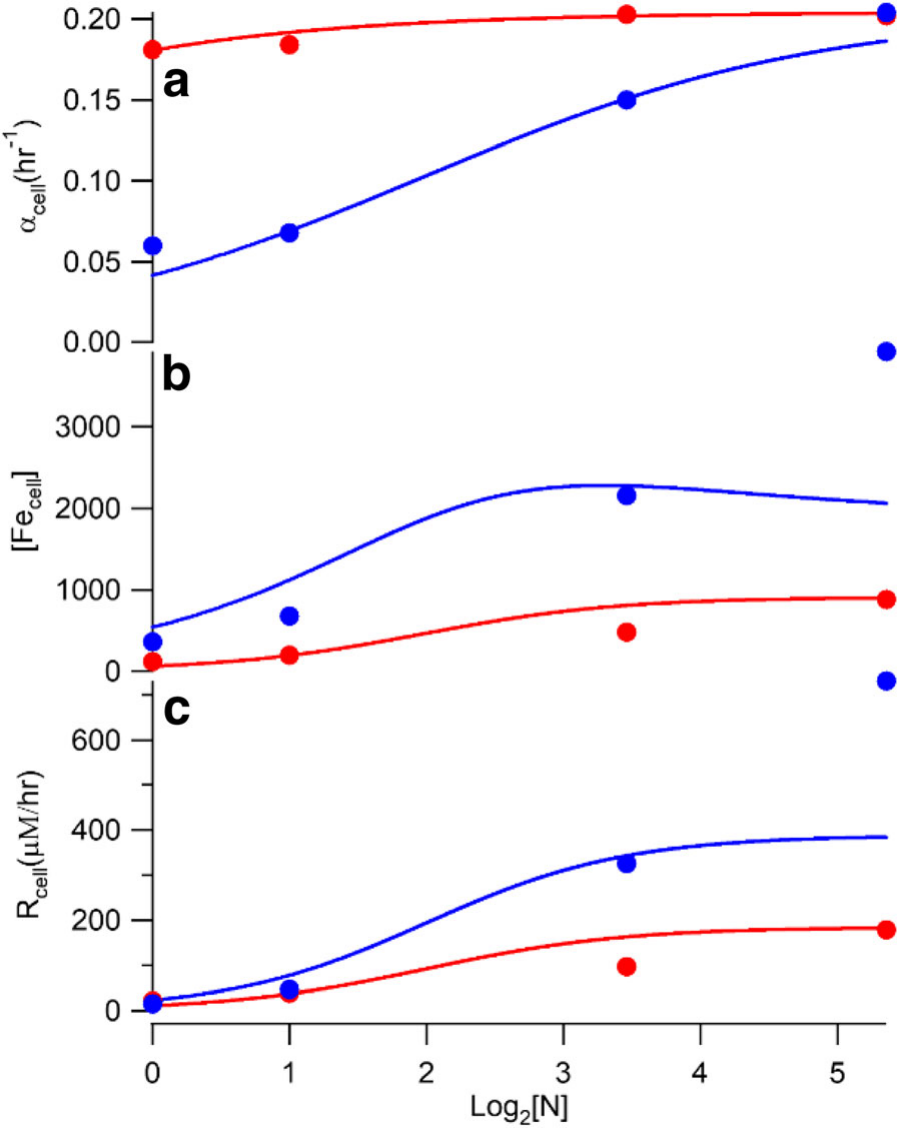
Plots of growth rate (**a**), cellular iron concentration (**b**), and the rate of iron import into the cell (**c**). Red circles and lines indicate data-based and simulated WT cells. Blue circles and lines indicate ΔΔ cells. Data-based values and corresponding simulation values are given in Table 1. For data points in this figure and in Fig. 3, the errors estimated in [34] from two determinations are within the marks. Overall errors may be higher

This error function normalized absolute differences between simulations and data to the average of simulation and data values. This formulation weighed the error associated with each datapoint evenly without regard to the magnitude of the point. Best-fit *α*_max_ and *K*_*α*_values are given in Table 2, and plots of *α*_*cell*_ are shown in Fig. 2a. The simulated growth rates of WT and ΔΔ cells increased as the concentration of iron in the medium [N] increased, mirroring the experimental growth rates with acceptable fidelity (apart from the point associated with ΔΔ cells at [N] = 41 μM). *Acceptable fidelity* is a qualitative term which means that simulations “trended” with the data – i.e. increasing when the data increased, decreasing when they decreased, and remaining flat when they remained flat. Due to the limited amount of data and our trial-and-error method of optimizing, this term is more appropriate than other more quantitative descriptors. The high iron concentration of ΔΔ cells grown with 40 μM ferric citrate is consistent with Mössbauer spectra which are very high-intensity. ΔΔ cells are iron-dysregulated and they import large amounts of iron, and the model developed here can only account for a portion of that accumulation. A more complex model could fit the point better but we opted to keep the model simple.

**Table 2:** Optimized parameters used in simulations

Parameter	Value (strain)	units	Sensitivity
*f*_*cyt*_	0.8	none	1.051
*f*_*mit*_	0.1	none	1.042
*f*_*vac*_	0.1	none	1.001
C_1_			
*R*_*cell-max*_	180 (WT)	μM hr.^−1^	1.021
	390 (ΔΔ)		1.049
*K*_*N*_	4	μM	1.062
*sens*	2	none	1.043
α_max_	0.204	hr^−1^	1.068
*K*_*α*_	0.13(WT)	hr^−1^	1.047
	3.9 (ΔΔ)		1.029
C_3_			
*R*_*cyt*_	230 (WT)	μM hr.^−1^	1.011
	480 (ΔΔ)		1.024
*k*_*mit(C3)*_	2.8(WT)	hr^−1^	1.001
	1.6 (ΔΔ)		1.000
*R*_*vac-max*_	1140	μM hr.^−1^	1.000
*K*_*vac*_	11	μM	1.001
*nvac*	3	none	1.001
C_4_			
*R*_*cia-max*_	56	μM hr.^−1^	1.001
*K*_*cia*_	3.8	μM	1.000
*ncia*	3	none	1.000
C_9_			
*k*_*mit*_	5.5(WT)	hr^−1^	1.000
	1.2 (ΔΔ)		1.000
*R*_*isu-max*_	180	μM hr.^−1^	1.005
*K*_*isu*_	220	μM	1.021
*nisu*	2.3	none	1.106
*k*_*vp*_	1.10 × 10^−7^ (WT)	μM^1-nvp^ hr.^−1^	1.000
	2.37 × 10^− 7^ (ΔΔ)		1.000
*nvp*	2.4	none	1.001
*k*_*23*_	5.2	hr^−1^	1.000
[FS]_sp_	370	μM	1.000
*n23*	1.6	none	1.000
*k*_*mp*_	0.09	μM^−1^ h^−1^	1.013
*k*_*O2*_	25	hr^−1^	1.008
*k*_*res*_	9	μM^−1^ h^− 1^	1.010

In the C_1_ model, all iron in the cell is considered to be a single component called *Fe*_*cell*_. The concentration [Fe_cell_] is a function of moles (*n*_*Fecell*_) and volume *V*_*cell*_, namely [Fe_cell_] = *n*_*Fecell*_/*V*_*cell*_. Since the cell is growing as chemistry is occurring, the time-dependent change of [Fe_cell_] is given by the partial derivative

4 $$ \left\{\begin{array}{l}\frac{d\left[{Fe}_{cell}\right]}{dt}={\left.\frac{\partial \left[{Fe}_{cell}\right]}{\partial {n}_{Fe cell}}\cdot \frac{dn_{Fe cell}}{dt}\right|}_{\mathrm{constant}\ \mathrm{V}}+{\left.\frac{\partial \left[{Fe}_{cell}\right]}{\partial {V}_{cell}}\cdot \frac{dV_{cell}}{dt}\right|}_{\mathrm{constant}\ \mathrm{n}}\\ {}\frac{d\left[{Fe}_{cell}\right]}{dt}={\left.\frac{1}{V_{cell}}\frac{dn_{Fe cell}}{dt}\right|}_{\mathrm{constant}\ \mathrm{V}}-{\left.\frac{n_{Fe cell}}{{\left({V}_{cell}\right)}^2}\cdot \frac{dV_{cell}}{dt}\right|}_{\mathrm{constant}\ \mathrm{n}}\\ {}\frac{d\left[{Fe}_{cell}\right]}{dt}={\left.\frac{d\left[{Fe}_{cell}\right]}{dt}\right|}_{\mathrm{constant}\kern0.5em \mathrm{V}}-{\left.\frac{1}{V_{cell}}\cdot \frac{dV_{cell}}{dt}\left[{Fe}_{cell}\right]\right|}_{\mathrm{constant}\ \mathrm{n}}\\ {}\frac{d\left[{Fe}_{cell}\right]}{dt}={R}_{cell}-{\alpha}_{cell}\cdot \left[{Fe}_{cell}\right]\end{array}\right\}. $$

The first term on the right-hand-side of the last equation of (4) describes the rate of iron import at constant volume ($N\overset{R_{cell}}{\to }{Fe}_{cell}$) – i.e. for chemistry occurring in a no-growth cell. The second term reflects dilution due to the growth of cells at constant moles of *Fe*_*cell*_ - .i.e. for a growing cell devoid of chemistry. Yeast cells lack iron exporters (unlike mammalian cells that contain ferroportin) and no export process is known. Under the *expanding-steady-state* condition, as would exist for a population of exponentially growing cells, the left-hand-side of (4) equals zero, [Fe_cell_] is constant and the import rate *R*_*cell*_ equals the dilution rate,

5 $$ {R}_{cell}={\alpha}_{cell}\cdot \left[{Fe}_{cell}\right]. $$

[Fe_cell_] was measured in WT and ΔΔ cells grown under the four concentrations of [N] [34], and the product of this and corresponding *α*_*cell* − *dat*_ values afforded the “data-based” *R*_*cell-dat*_ values listed in Table 1. These values are shown as the circles in Fig. 2c. Plots of [Fe_cell_] vs. log_2_[N] are given in Fig. 2b.

We next assigned a rate-law expression to *R*_*cell*_ that depended solely on [N], such that a continuous *R*_*cell-sim*_ function could be generated at all [N]. The iron-importer on the plasma membrane of yeast cells is saturatable by nutrient iron [35], and so we assigned the rate-law for *R*_*cell-sim*_ to the Hill function

6 $$ {R}_{cell- sim}=\frac{R_{\mathrm{cell}\hbox{-} \max }{\left[N\right]}^{sens}}{{K_N}^{sens}+{\left[N\right]}^{sens}} $$

where *sens* is a Hill coefficient allowing for cooperative iron import. *R*_*cell-sim*_ was optimized by minimizing an ERR function similar to Eq. (3). The resulting optimized *R*_*cell-sim*_ simulation parameters are given in Table 2. The *R*_*cell-sim*_ equation was used to generate an ODE (based on the last equation in (4)) that could be used in kinetic modeling (see Additional file 1: Equation S1). However, the current study focuses on the expanding steady-state condition, and so ODEs S1 and S2 were solved at infinitely long times for [N] ranging from 1 to 41. Plots of steady-state *R*_*cell-sim*_ vs. log_2_[N] are shown in Fig. 2 bottom panel. As expected, the simulated rate of iron import increased in both WT and ΔΔ cells as the concentration of iron in the medium increased, with higher rates for ΔΔ cells since they accumulate more iron. The [N]-dependent increase in iron import rate is counterbalanced by the [N]-dependent increase in cell growth rate.

### Development of the C_3_ model

In the C_3_ model, cell volume was divided into mitochondria, vacuoles and all remaining compartments, such that

7 $$ {V}_{cell}={V}_{cyt}+{V}_{mit}+{V}_{vac}. $$

Here, “*cyt*” refers to cytosol *plus* all organelles besides mitochondria and vacuoles; there is insufficient published information to justify subdividing cyt into additional cellular compartments. This collective compartment includes the iron content of the nucleus which contains a significant number of [Fe_4_S_4_] containing proteins [36]. Topologically, cyt was treated as though it was exclusively cytosol i.e. surrounding mitochondria and vacuoles and being surrounded by the plasma membrane.

Each cellular compartment in the C_3_ model was presumed to contain a single iron species, called *Fe*_*cyt*_, *Fe*_*mit*_, and *Fe*_*vac*_. The conservation of matter requires that

8 $$ \left[{Fe}_{cell}\right]={f}_{cyt}\cdot \left[{Fe}_{cyt}\right]+{f}_{mit}\cdot \left[{Fe}_{mit}\right]+{f}_{vac}\cdot \left[{Fe}_{vac}\right] $$

where *f*_*cyt*_, *f*_*mit*_, and *f*_*vac*_ are fractional volumes e.g. *f*_*mit*_ = *V*_*mit*_/*V*_*cell*_. In an expanding steady-state, these fractional volumes will be constant such that

9 $$ \frac{d\left[{Fe}_{cell}\right]}{dt}={f}_{cyt}\cdot \frac{d\left[{Fe}_{cyt}\right]}{dt}+{f}_{mit}\cdot \frac{d\left[{Fe}_{mit}\right]}{dt}+{f}_{vac}\cdot \frac{d\left[{Fe}_{vac}\right]}{dt}. $$

All of the derivative terms in (9) are zero in an expanding steady state. For the C_3_ model, *N* is imported into the cytosol forming *Fe*_*cyt*_ ($N\overset{R_{cyt}}{\to }{Fe}_{cyt}$). Some *Fe*_*cyt*_ is imported into mitochondria (${Fe}_{cyt}\overset{R_{mit}}{\to }{Fe}_{mit}$) and some into vacuoles (${Fe}_{cyt}\overset{R_{vac}}{\to }{Fe}_{vac}$). The rest remains in cyt. Based on this scheme, the time-dependent changes of the concentrations of the Fe species in each region are

10 $$ \left\{\begin{array}{l}\frac{d\left[{Fe}_{cyt}\right]}{dt}={R}_{cyt}-{R}_{mit}-{R}_{vac}-\frac{1}{V_{cyt}}\frac{dV_{cyt}}{dt}\left[{Fe}_{cyt}\right]\\ {}\frac{d\left[{Fe}_{mit}\right]}{dt}=\frac{f_{cyt}}{f_{mit}}{R}_{mit}-\frac{1}{V_{mit}}\frac{dV_{mit}}{dt}\cdot \left[{Fe}_{mit}\right]\\ {}\frac{d\left[{Fe}_{vac}\right]}{dt}=\frac{f_{cyt}}{f_{vac}}{R}_{vac}-\frac{1}{V_{vac}}\frac{dV_{vac}}{dt}\cdot \left[{Fe}_{vac}\right]\end{array}\right\}. $$

Equation (10) follows from the proposed mechanism in which iron first flows into the cytosol and then cytosolic iron *Fe*_*cyt*_ flows into mitochondria and vacuoles. Volume ratios in the second and third equations of (10) are required to conserve mass as *Fe*_*cyt*_ moves from one region to another. Under an expanding steady-state, the left-hand-sides of (10) equal zero and

11 $$ \left\{\begin{array}{l}{R}_{cyt}={R}_{mit}+{R}_{vac}+\frac{1}{V_{cyt}}\frac{dV_{cyt}}{dt}\left[{Fe}_{cyt}\right]\\ {}{R}_{mit}=\frac{1}{V_{cyt}}\frac{dV_{mit}}{dt}\cdot \left[{Fe}_{mit}\right]\\ {}{R}_{vac}=\frac{1}{V_{cyt}}\frac{dV_{vac}}{dt}\cdot \left[{Fe}_{vac}\right]\end{array}\right\}. $$

The growth rate of each cellular region will equal the growth rate of the cell multiplied by the fractional volume of that compartment,

12 $$ \left\{\begin{array}{l}\frac{dV_{cyt}}{dt}={f}_{cyt}\frac{dV_{cell}}{dt}\\ {}\frac{dV_{mit}}{dt}={f}_{mit}\frac{dV_{cell}}{dt}\\ {}\frac{dV_{vac}}{dt}={f}_{vac}\frac{dV_{cell}}{dt}\end{array}\right\}. $$

Substituting (12) into (11), and using (1) and (8) affords

13 $$ \left\{\begin{array}{l}{R}_{cyt}={R}_{mit}+{R}_{vac}+{\alpha}_{cell}\cdot \left[{Fe}_{cyt}\right]\\ {}{R}_{mit}=\frac{V_{mit}}{V_{cyt}}{\alpha}_{cell}\cdot \left[{Fe}_{mit}\right]\\ {}{R}_{vac}=\frac{V_{vac}}{V_{cyt}}{\alpha}_{cell}\cdot \left[{Fe}_{vac}\right]\end{array}\right\}. $$

Published fractional volumes were used to help solve these equations. The cellular content of fermenting exponentially growing, nonbudding *S. cerevisiae* was reconstructed in 3D, and volume fractions were determined [37]. Mitochondria and vacuoles occupied 1.7% and 5.8% of cell volume, respectively. Another study reported that the same two organelles occupied 1.6 and 7.8%, respectively [38]. In a third study, vacuoles in yeast strain W303 (the same as used in our studies) accounted for 10% of cell volume [39]. And in *respiring* yeast cells, mitochondrial volume was 10%–12% of cell volume [40]. Since the model developed here is of iron trafficking in respiring W303 yeast cells, we assumed *f*_*mit*_ = 0.1, *f*_*vac*_ = 0.1, and *f*_*cyt*_ = 0.8.

The relationships given in (13) are connected to (5). Substituting the last two equations of (13) into the first, and then simplifying and comparing to (5) affords the relationship

14 $$ {R}_{cyt}=\frac{1}{f_{cyt}}{R}_{cell}. $$

This equation connects C_1_ and C_3_ models. The rate of iron import into cyt (*R*_*cyt*_) equals the data-based rate of Fe import into the cell (*R*_*cell*_) divided by the volume fraction *f*_*cyt*_. These rates describe the change of iron *concentrations* within the cell or cytosol, not the change in the number of moles of *N* imported. Since *V*_*cyt*_ < *V*_*cell*_, [Fe_cyt_] will increase faster than [Fe_cell_], in proportion to the ratio *V*_*cell*_*/V*_*cyt*_. This is true even though the same number of moles of iron per unit time is imported. The rate-law expression for *R*_*cyt-sim*_ should also involve a Hill expression, with the same *K*_*N*_ and *sens* as in (6) but with a maximal velocity that is 1.25-times (1/*f*_*cyt*_) faster.

The C_3_ model could not be solved fully until [Fe_cell_] was separated into [Fe_cyt_], [Fe_mit_], and [Fe_vac_] components for each of the 8 growth/strain conditions investigated. To do this, we relied on the conservation-of-matter Eq. (8), published MB spectra, and on iron concentrations for WT and ΔΔ cells and organelles [34, 41]. The spectra were separated into contributions from the eight iron-containing components specified by the C_9_ model. Then we combined particular components into cytosol, mitochondria, or vacuoles locations (as defined by the model of Fig. 1). Finally, we summed the iron concentrations for all of the components assigned to each compartment to afford our best data-based estimates of [Fe_cyt_], [Fe_mit_], and [Fe_vac_]. Results are given in Table 1.

### Development of the C_9_ model

Before explaining how MB spectra were decomposed, we introduce the components of the C_9_ model. Component *C* represents a NHHS Fe^II^ complex presumed to be present in the cytosol. This component can move into vacuoles and mitochondria, but it can also stay in cyt and react to form component *CIA* (the Cytosolic Iron Sulfur Assembly machine), a second cytosolic iron species. *CIA* represents the sum of the ISCs and low-spin Fe^II^ heme groups in this collective compartment. Numerous ISCs are found in the cytosol and nucleus [37, 42], justifying the inclusion of *CIA* in the model. *FM* represents the pool of NHHS Fe^II^ ions in mitochondria, *FS* represents ISCs and heme centers in the organelle, and *MP* refers to mitochondrial nanoparticles. Components *FM*, *FS*, and *MP* have all been characterized experimentally. *F2* and *F3* are NHHS Fe^II^ and Fe^III^ species in vacuoles, and *VP* represent vacuolar nanoparticles; they have also been characterized experimentally [9, 43]. When *C* enters the vacuoles, this component becomes *F2* some of which oxidizes to *F3*. Some *F3* converts into *VP*. When *C* enters mitochondria, it converts into *FM*, which serves as feedstock for *FS*. *FS* metal centers are viewed as being installed into the respiratory complexes on the inner membrane, which then catalyze the reduction of *O*_*2*_ to water. *FM* can also react with model component *O*_*2*_ in the matrix to generate *MP* and ROS. ROS exhibits the exact behavior of *MP* so is not formally included in the model.

### Decomposing Mössbauer features into modeling components

MB spectroscopy detects all of the ^57^Fe in samples. However, resolution is limited so the spectra under consideration were subdivided into just four groups of iron centers, including NHHS Fe^III^, NHHS Fe^II^, the central doublet (CD), and Fe^III^ oxyhydroxide nanoparticles. The CD represents [Fe_4_S_4_]^2+^ clusters and low-spin Fe^II^ heme centers; the two cannot be resolved. Other minor spectral features (HS Fe^II^ heme groups and [Fe_2_S_2_] clusters) can be resolved and quantified, but we decided to bundle them with the CD since they are not individually represented in the model. The absolute concentrations associated with each group were obtained by multiplying the associated percentages by [Fe_cell_]. The conservation of mass requires that

15 $$ \left\{\begin{array}{l}\left[{Fe}_{cell}\right]=\left[{Fe^{II}}_{cell}\right]+\left[{CD}_{cell}\right]+\left[{NP}_{cell}\right]+\left[{Fe^{II I}}_{cell}\right]\\ {}\left[{Fe}_{mit}\right]=\left[{Fe^{II}}_{mit}\right]+\left[{CD}_{mit}\right]+\left[{NP}_{mit}\right]+\left[{Fe^{II I}}_{mit}\right]\end{array}\right\}. $$

These MB features were assigned to the following combinations of modeling components.

16 $$ \left\{\begin{array}{l}\left[{Fe^{II}}_{cell}\right]={f}_{cyt}\cdot \left[C\right]+{f}_{mit}\cdot \left[ FM\right]+{f}_{vac}\cdot \left[F2\right]\\ {}\left[{CD}_{cell}\right]={f}_{cyt}\cdot \left[ CIA\right]+{f}_{mit}\cdot \left[ FS\right]\\ {}\left[{NP}_{cell}\right]={f}_{mit}\cdot \left[ MP\right]+{f}_{vac}\cdot \left[ VP\right]\\ {}\left[{Fe^{II I}}_{cell}\right]={f}_{vac}\cdot \left[F3\right]\\ {}\left[{Fe^{II}}_{mit}\right]=\left[ FM\right]\\ {}\left[{CD}_{mit}\right]=\left[ FS\right]\\ {}\left[{NP}_{mit}\right]+\left[{Fe^{II I}}_{mit}\right]=\left[ MP\right]\end{array}\right\}. $$

Then these species were organized into the three cellular compartments by summing contributions as described by (17).

17 $$ \left\{\begin{array}{l}\left[{Fe}_{cyt}\right]=\left[C\right]+\left[ CIA\right]\\ {}\left[{Fe}_{mit}\right]=\left[ FM\right]+\left[ FS\right]+\left[ MP\right]\\ {}{Fe}_{vac}\Big]=\left[F2\right]+\left[F3\right]+\left[ VP\right]\end{array}\right\}. $$

The one component of the C_9_ model that could not be determined in this way with reasonable accuracy was component *C*. Thus, we relied on published reports to estimate the concentration of cytosolic NHHS Fe^II^. Petrat et al. [44] used a fluorescent chelator to quantify the concentration of labile iron in hepatocytes and liver endothelial cells at 5–7 μM, and we assumed similar values for iron-sufficient WT yeast cells. We further assumed that the concentration of cytosolic Fe^II^ increases with increasing nutrient iron concentrations, and that [C] in iron-sufficient ΔΔ cells is higher than in WT cells (because the absence of Mrs3/4 should block import of *C* into mitochondria). Within these constraints, we assigned the concentrations of *C* to those listed in Table 3 so as to minimize the ERR function when the other iron concentrations in the table were used. MB spectral decompositions, along with these relationships and assumptions, were sufficient to generate concentrations for all other modeling components (Table 3).

**Table 3:** Estimated concentrations (in μM) of the iron-containing components of the C_9_ model. For each entry, the top number is data-based while the bottom number is the corresponding simulated value. The sum of these concentrations, after multiplying each by their respective fractional volume, approximately equals [Fe_cell_]. The sum of the concentrations of each species located in each compartment (cyt, mitochondria, and vacuoles) approximately equals [Fe_cyt_], [Fe_mit_], and [Fe_vac_], respectively

[N]	[C]	[CIA]	[FM]	[FS]	[MP]	[F2]	[F3]	[VP]
WT								
1	2.5	92	60	320	0	60	0	0
	1.5	16	110	160	90	14	85	0
2	3	84	80	290	60	96	780	0
	2.7	79	190	380	62	43	590	2.6
11	4	250	140	380	30	320	1900	0
	5.6	210	430	730	71	250	540	320
41	5	380	210	480	0	450	4600	0
	6.0	220	470	750	75	290	5200	440
ΔΔ								
1	3	130	60	40	530	140	400	0
	2.6	310	3.6	0.8	580	2300	57	0.6
2	4	160	60	40	530	1300	3400	170
	4.3	470	100	350	140	180	4600	2000
11	8	340	85	300	300	950	11,000	6400
	8.6	340	150	330	70	1200	11.000	7500
41	10	280	110	560	80	1000	22,000	13,000
	9.2	280	140	260	71	1600	10,000	5700

#### Solving the C_3_ model

**Fig. 3:**
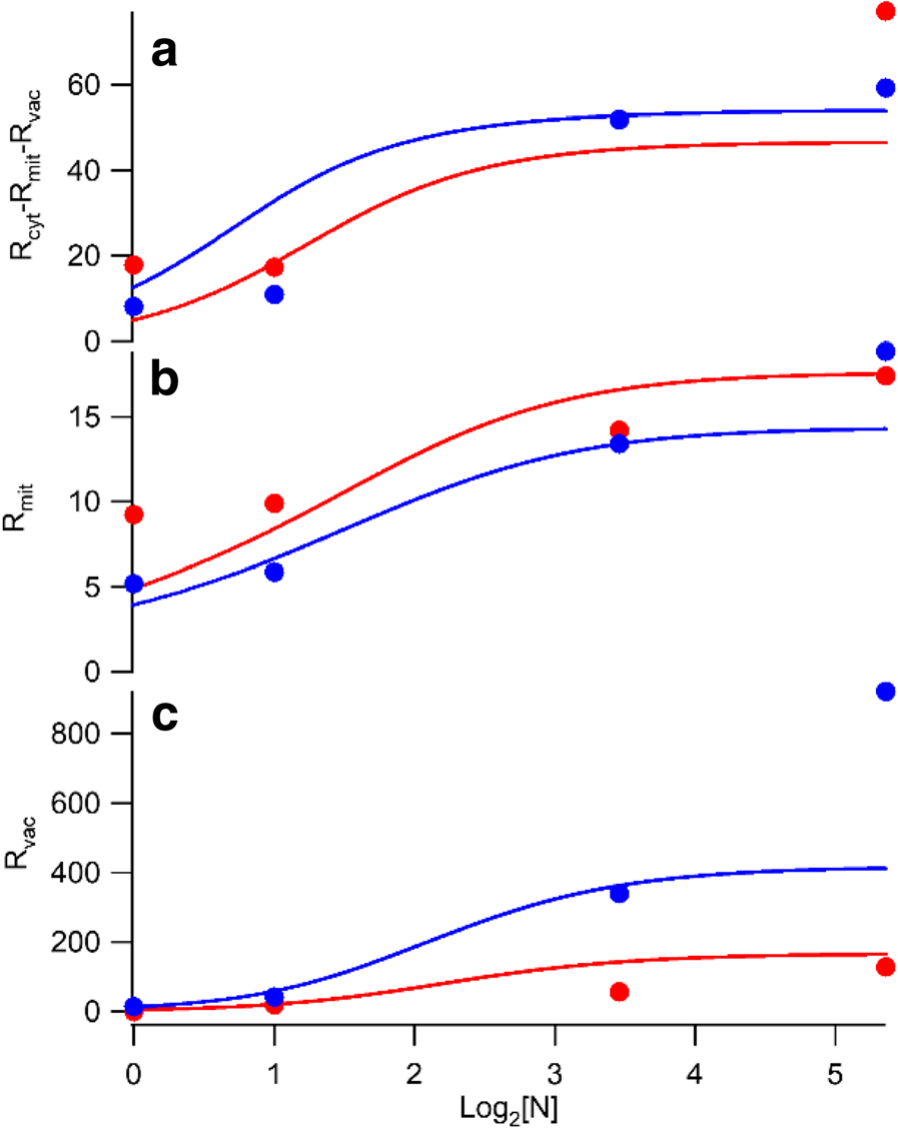
Rates of iron import into the cytosol *only* (**a**), into the mitochondria (**b**), and into vacuole (**c**) according to the C_3_ model. Color coding is the same as in Fig. 2

Once [Fe_cyt_], [Fe_mit_] and [Fe_vac_] were determined, we determined rates of import into each compartment, *R*_*cyt*_, *R*_*mit*_, and *R*_*vac*_ as defined by (13). Data-based import rates *R*_*cyt-dat*_, *R*_*mit-dat*_, and *R*_*vac-dat*_ for the 8 conditions are shown as circles in Fig. 3 and are tabulated in Table 1. The rate of iron import into “cyt” but not exported into mitochondria or vacuoles equals *R*_*cyt*_ – *R*_*mit*_ - *R*_*vac*_. According to these rates, iron flows faster into the cyt of ΔΔ cells, and slower into their mitochondria, relative to in WT cells. This makes sense because the absence of Mrs3/4 in ΔΔ cells should hinder *Fe*_*cyt*_ from entering mitochondria.

We next assigned rate-law expressions to *R*_*mit-sim*_ and *R*_*vac-sim*_. We considered two forms for rate-laws, namely a mass-action form *R*_*i*_ = *k*_*i*_[C]^*n*^ and a Hill form *V*_*i*_[C]^*n*^/{*K*_*M*_+[C]^*n*^} where [C] indicates the concentration of *C* as defined by the C_9_ model components. The latter form was used only when the simpler mass-action form was unable to generate reasonable simulations of the relevant data-based rates. The simple mass-action form was acceptable for *R*_*mit-sim*_ whereas *R*_*vac-sim*_ required a Hill term. The terms were optimized using an ERR function. The following rate-law expressions were ultimately selected.

18 $$ \left\{\begin{array}{l}{R}_{mit- sim}={k}_{mit}\left[C\right]\\ {}{R}_{vac- sim}=\frac{R_{vac-\max }{\left[C\right]}^{nvac}}{K_{vac}^{nvac}+{\left[C\right]}^{nvac}}\end{array}\right\}. $$

One potentially confusing aspect of solving this system was that we used *C* (a component of the C_4_ and C_9_ models) rather than *Fe*_*cyt*_ (a component of the C_3_ model) as substrate for these processes. This was done so that the resulting rate-constant k_mit_ and R_vac-max_ would not change when solving the C_9_ model. Had we used *Fe*_*cyt*_ instead of *C* as substrate in the C_3_ model, *k*_*mit*_ and *R*_*vac-max*_ would have been too small for the C_4_ and C_9_ models in which [Fe_cyt_] = [C] + [CIA]. The rate of iron flowing into mitochondria depends only on [C], not on [C] + [CIA]. Optimized *R*_*mit-sim*_ and *R*_*vac-sim*_ values were used along with *R*_*cyt-sim*_ (obtained from the C_1_ model), to construct a full set of ODEs (Additional file 1: Equations S3–S5) describing the C_3_ model. Once combined in this way, all of the parameters associated with the three rates *R*_*cyt-sim*_, *R*_*mit-sim*_, and *R*_*vac-sim*_ were re-optimized against data-based rates using an ERR function. To do this, each parameter was increased and decreased by 10% as all other parameters were fixed; candidate values that lowered ERR were then fixed as the next parameter on the list was varied. The process was repeated for a second round except that each parameter was adjusted ±5%. In the third and final round, each parameter was adjusted ±1%. The final plots are shown in Fig. 3.

### The C_4_ model

We next solved the C_4_ model which was identical to C_3_ except that [Fe_cyt_] was separated into [CIA] and [C] components. To obtain [CIA], we subtracted the values of [C] given in Table 3 from [Fe_cyt_], resulting in the *CIA* concentrations listed in Table 3. These values were multiplied by *α*_*cell*_ to generate *R*_*cia-dat*_. We assumed a Hill expression to generate an *R*_*cia-sim*_ function that minimized differences with *R*_*cia-dat*_ with acceptable fidelity.

#### Solving the C_9_ model

At this point, the C_9_ model could be solved. The derivative of (17) is

19 $$ \left\{\begin{array}{l}\frac{d\left[{Fe}_{cyt}\right]}{dt}=\frac{d\left[C\right]}{dt}+\frac{d\left[ CIA\right]}{dt}\\ {}\frac{d\left[{Fe}_{mit}\right]}{dt}=\frac{d\left[ FM\right]}{dt}+\frac{d\left[ FS\right]}{dt}+\frac{d\left[ MP\right]}{dt}\\ {}\frac{d\left[{Fe}_{vac}\right]}{dt}=\frac{d\left[F3\right]}{dt}+\frac{d\left[ VP\right]}{dt}\end{array}\right\}. $$

According to the mechanism of Fig. 1, bottom panel, the rates of change in the concentrations of the two cyt iron species are

20 $$ \left\{\begin{array}{l}\frac{d\left[C\right]}{dt}={R}_{cyt}-{R}_{mit}-{R}_{vac}-{R}_{cia}-{\alpha}_{cell}\left[C\right]\\ {}\frac{d\left[ CIA\right]}{dt}={R}_{cia}-{\alpha}_{cell}\left[ CIA\right]\end{array}\right\}. $$

Adding the two equations of (20) affords the first equations of (19) and (10). The rate of change of the concentrations of the iron-containing species in the mitochondria is given by (21),

21 $$ \left\{\begin{array}{l}\frac{d\left[ FM\right]}{dt}=\frac{V_{cyt}}{V_{mit}}{R}_{mit}-{R}_{isu}-{R}_{mp}-{\alpha}_{cell}\left[ FM\right]\\ {}\frac{d\left[ FS\right]}{dt}={R}_{isu}-{\alpha}_{cell}\left[ FS\right]\\ {}\frac{d\left[ MP\right]}{dt}={R}_{mp}-{\alpha}_{cell}\left[ MP\right]\end{array}\right\}. $$

Summing the equations of (21) affords the second equations of (19) and (10). Similarly for vacuoles,

22 $$ \left\{\begin{array}{l}\frac{d\left[F2\right]}{dt}=\frac{V_{cyt}}{V_{vac}}{R}_{vac}-{R}_{23}-{\alpha}_{cell}\left[F2\right]\\ {}\frac{d\left[F3\right]}{dt}={R}_{23}-{R}_{vp}-{\alpha}_{cell}\left[F3\right]\\ {}\frac{d\left[ VP\right]}{dt}={R}_{vp}-{\alpha}_{cell}\left[ VP\right]\end{array}\right\}. $$

Summing the equations of (22) affords the third equations of (19) and (10). Thus, the ODE system for the iron-components of the C_9_ model “collapses” down to that of the C_3_ model when the components of the three regions are summed appropriately. In an expanding steady state, the left-hand-sides of (20), (21), and (22) equal zero such that

23 $$ \left\{\begin{array}{l}{R}_{cyt}={R}_{mit}+{R}_{vac}+{R}_{cia}+{\alpha}_{cell}\left[C\right]\\ {}{R}_{mit}=\frac{V_{mit}}{V_{cyt}}\left({R}_{isu}+{R}_{mp}+{\alpha}_{cell}\left[ FM\right]\right)\\ {}{R}_{vac}=\frac{V_{vac}}{V_{cyt}}\left({R}_{23}+{\alpha}_{cell}\left[F2\right]\right)\\ {}{R}_{cia}={\alpha}_{cell}\left[ CIA\right]\\ {}{R}_{isu}={\alpha}_{cell}\left[ FS\right]\\ {}{R}_{vp}={\alpha}_{cell}\left[ VP\right]\\ {}{R}_{23}={R}_{vp}+{\alpha}_{cell}\left[F3\right]\\ {}{R}_{mp}={\alpha}_{cell}\left[ MP\right]\end{array}\right\}. $$

Data-based and simulation-based values of *R*_*cyt*_, *R*_*mit*_, *R*_*vac*_, and *R*_*cia*_ have already been obtained. Using the experimental values of *α*_*cell*_ and the values of model-component concentrations listed in Table 3, we constructed data-based rates for the formation of each C_9_ component using data from the 4 nutrient conditions in WT and ΔΔ cells (Table 4). *R*_*vp-dat*_ was then used along with *α*_*cell*_ and [F3] to generate *R*_*23-dat*_ as defined in (23). The next step was to assign a rate-law expression to each of the remaining rates associated with the C_9_ model as listed in (23) – expressions that depended solely on other C_9_ components. Once assigned, a system of ODEs could be defined in these terms (Additional file 1: Equations S6–S14) and integrated numerically to afford our final simulations.

**Table 4:** Rates of formation of each component of the C_9_ model, for different strains and nutrient concentrations. Data-based rates are the top entries; simulated rates are bottom entries

[N]	*R*_*C*_	*R*_*CIA*_	*R*_*isu*_	*R*_*F3*_	*R*_*VP*_	*R*_*FM*_	*R*_*FS*_	*R*_*MP*_
WT								
1	0.45	17	52	0	0	11	58	0
	0.26	3.0	29	15	0	19	29	16
2	0.55	15	53	140	0	15	53	11
	0.52	15	73	110	0.5	36	73	12
11	0.81	50	78	390	0	29	78	6.1
	1.1	43	150	910	65	87	150	14
41	1.0	76	97	930	0	42	91	0
	1.2	44	150	1000	89	95	150	15
ΔΔ								
1	0.12	8.0	2.4	24	0	3.6	2.4	32
	0.11	13	0.014	0.75	0	0.13	0.01	24
2	0.28	11	2.7	230	12	4.1	2.7	36
	0.29	33	27	320	140	7.2	27	7.1
11	1.2	51	45	1600	960	13	45	45
	1.3	52	50	1600	1100	22	50	11
41	1.9	57	110	4500	2600	22	110	15
	1.7	52	48	2000	1100	27	48	13

We first assigned rate-law expressions for the remaining C_9_ components that did not involve O_2_, namely *R*_*vp*_ and *R*_*isu*_. The expressions *k*_*vp*_⋅[F3] and *k*_*isu*⋅_[FM] were sufficient to simulate *R*_*vp-dat*_ and *R*_*isu-dat*_ with acceptable fidelity. The simple rate-law *R*_*23-sim*_ = *k*_*23*⋅_[F2] was unable to simulate the data. The problem was that cells grown under low-iron conditions have an unusually high concentration of NHHS Fe^II^, only a small percentage of which can be assigned to *FM* in mitochondria. Under these conditions, it seemed unlikely that this Fe^II^ could be cytosolic, as there should be low concentrations of [C] (as given in Table 3). The only remaining option (in our model) that could account for the extra Fe^II^ was component *F2* in vacuoles. As cells become iron-sufficient, this effect disappears as [F3] increases. We presumed that the extra *F2* converted into *F3* under these conditions. To coordinate this behavior with increasing cellular iron-sufficiency, we incorporated a Reg_+FS_ function into the *R*_*23-sim*_ rate-law expression, as we have done previously [17]. In summary, the following rate-law expressions were used in solving the C_9_ model.

24 $$ \left\{\begin{array}{l}{R}_{cia}=\frac{R_{cia-\max }{\left[C\right]}^{ncia}}{K_{cia}^{ncia}+{\left[C\right]}^{ncia}}\\ {}{R}_{isu}=\frac{R_{isu-\max }{\left[ FM\right]}^{nisu}}{K_{isu}^{nisu}+{\left[ FM\right]}^{nisu}}\\ {}{R}_{vp}={k}_{vp}{\left[F3\right]}^{nvp}\\ {}{R}_{23}={k}_{23}\left[F2\right]\left(\frac{1}{1+{\left(\frac{{\left[ FS\right]}_{sp}}{\left[ FS\right]}\right)}^{n23}}\right)\end{array}\right\}. $$

The Reg_+FS_ function is the parenthetical term associated with *R*_*23*_ in Eq. (24). It may be viewed as a valve that regulates the rate by which *F2* converts to *F3*. The valve is almost fully open (value near to 1) when [FS] is much greater than the set-point concentration [FS]_sp_, and is nearly closed (value near to 0) when [FS] is much less than [FS]_sp_.

### Effect of O_2_

Oxygen plays a critical role in the C_9_ model as it reacts with *FM* to generate *MP*. O_2_ is constantly diffusing into the matrix (in accordance with rate *R*_*O2*_) and is reduced to H_2_O by cytochrome c oxidase on the inner membrane. We used [FS] as a proxy for oxidase activity such that the rate of respiration (*R*_*res*_) was assumed to be proportional to both [FS] and [O_2_]. Collectively, these processes determine the dissolved O_2_ concentration in the matrix, as described by

25 $$ \frac{d\left[{O}_2\right]}{dt}={R}_{O2}-{R}_{mp}-{R}_{res}-{\alpha}_{cell}\left[{O}_2\right]. $$

Under an expanded steady-state condition

26 $$ {R}_{O2}={R}_{mp}+{R}_{res}+{\alpha}_{cell}\left[{O}_2\right]. $$

R_O2_ was presumed to be proportional to the *difference* in the O_2_ concentration in the cytosol (called [O_2_]_cyt_ – assumed to be fixed at 100 μM) and the concentration of O_2_ in the matrix ([O_2_]). With rate-law expressions included, (26) becomes

27 $$ {k}_{O2}\left({\left[{O}_2\right]}_{cyt}-\left[{O}_2\right]\right)={k}_{mp}\left[ FM\right]\left[{O}_2\right]+{k}_{res}\left[ FS\right]\left[{O}_2\right]+{\alpha}_{cell}\left[{O}_2\right]. $$

Rearrangement yields

28 $$ \left[{O}_2\right]={\left[{O}_2\right]}_{cyt}\frac{k_{O2}}{k_{O2}+{k}_{mp}\left[ FM\right]+{k}_{res}\left[ FS\right]+{\alpha}_{cell}}. $$

Since all numbers in (28) are positive, the term in the numerator serves to increase [O_2_] while those of the denominator serve to decrease it. [FM], [FS], and α_cell_ are controlled by other aspects of the model, and so those parameters were not altered in order to generate the behavior desired for [O_2_] vs [N] in WT vs. ΔΔ cells. This behavior was essentially controlled by the three unassigned parameters, *k*_*O2*_, *k*_*res*_, and *k*_*mp*_ contained in Eq. (28).

One major objective for this study was to explain how ΔΔ mitochondria transition from a diseased state (dominated by nanoparticles, *MP* in the model) when cells are grown in low-iron media, to a healthy state (dominated by ISCs and heme centers, *FS* in the model) when they are grown in high-iron media. We also wanted WT mitochondria to be healthy regardless of the iron concentration in the growth medium. The major molecular-level differences between WT and ΔΔ cells are the rates at which iron enters mitochondria (*R*_*mit*_) and cells (*R*_*cyt*_), and the growth rates – all of which have been set by solving the simpler versions of the model. The key to achieving the desired behavior, according to our model, was to vary the concentration of O_2_ in the matrix. In WT mitochondria, [O_2_] should be low at all [N] whereas in ΔΔ mitochondria, [O_2_] should be high at low [N] and low at high [N]. We needed to generate an abrupt decline of [O_2_] in ΔΔ mitochondria as [N] increases while keeping [O_2_] low in WT mitochondria at all [N]. *And* we needed to make this happen only by adjusting *k*_*O2*_, *k*_*res*_, and *k*_*mp*_.

The [O_2_] concentration in the matrix has not been measured directly. We estimated [O_2_] to be in the ballpark of 1–10 μM for iron-sufficient WT mitochondria as this value is similar to the *K*_*M*_ for O_2_ reduction by cytochrome c oxidase [45]. We had [MP] vs. [N] data that could be used to help optimize these parameters (especially *k*_*mp*_), but they were insufficient.

We also considered the known behavior of Yfh1Δ cells, which we have explained using a similar model [17]. Yfh1Δ mitochondria contain excessive levels of nanoparticles. The previous model explained the excessive nanoparticles as being due to a lack of *FS* (respiratory complexes), which allows for O_2_ to diffuse into the matrix, react with *FM*, and generate *MP*. This behavior (obtained by setting *R*_*isu*_ = 0) provided another constraint on possible solutions for the current problem. Another consideration was that respiring WT cells grown at all [N] do not accumulate *MP* in their mitochondria.

After extensive trials, we obtained values of *k*_*O2*_, *k*_*res*_, and *k*_*mp*_ (listed in Table 2) that generated the best overall behavior. However, despite our efforts to satisfy all of these constraints, we could not completely eliminate the formation of *MP* in WT mitochondria while also having *MP* accumulate at high levels in Yfh1Δ mitochondria. Two additional changes were required to do this, namely increasing *k*_*mit*_ of WT cells 2-fold and decreasing *k*_*mit*_ of ΔΔ cells 1.3-fold, both relative to the values obtained by solving the simpler C_3_ model. The adjustment of *k*_*mit*_ in ΔΔ cells was minor whereas the adjustment for WT cells implies that the concentration of iron in WT mitochondria is actually 2-fold higher than given by the data used for simulations.

In summary, solving the C_9_ model while achieving the desired behavior with O_2_ required that we increase *k*_*mit*_ of WT cells 2-fold and decrease k_mit_ of ΔΔ cells 1.3-fold relative to values obtained in the C_3_/C_4_ models. This explains the different values of *k*_*mit*_ in Table 2. The faster import rate from cytosol into the mitochondria for the C_9_ model also caused a slight decline of cytosolic iron ([C] and [CIA]).

### Final optimization and sensitivity analysis

Once each parameter was optimized individually as described above, we re-optimized the entire system by changing one component at a time while holding the others fixed, as described above. For the C_1_, C_3_/C_4_, and C_9_ model variants, the best-fit ERR values were 0.32, 0.39, and 0.72, respectively. A sensitivity analysis was performed for each parameter by taking the average of the ±1% ERR values, and normalizing the average to the optimal ERR for that parameter [17]. This procedure is calculated using the equation

29 $$ \frac{ERR_{opt+1\%}+{ERR}_{opt-1\%}}{2\cdot {ERR}_{opt}}. $$

Highest sensitivity values (Table 2) indicate parameters with the greatest impact on the overall fit of the model; *nisu* (Hill coefficient for ISC assembly), α_max_ (growth rate), *K*_*N*_ (the Michaelis-Menten constant for nutrient iron import), and *f*_*cyt*_ (fractional cytosol volume) were the most sensitive.

**Fig. 4:**
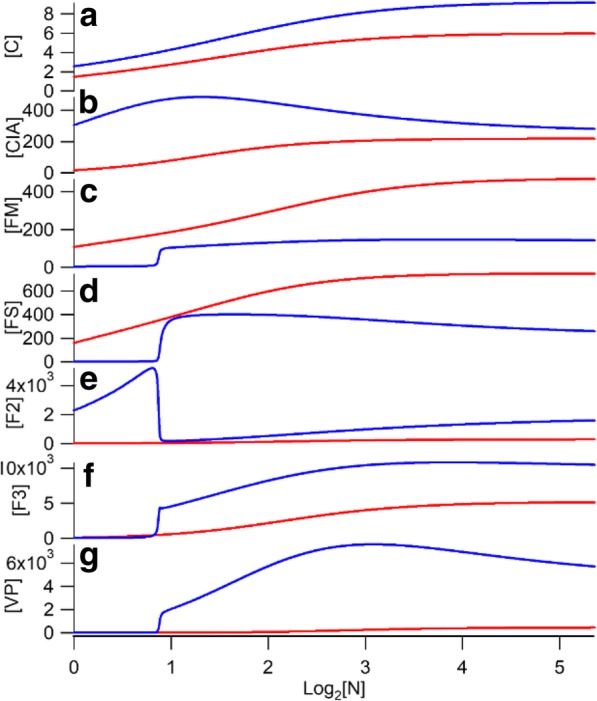
Simulated concentrations of the iron-containing components in the C_9_ version of the model as a function of nutrient iron concentration (in μM). Blue, ΔΔ cells; Red, WT cells. **a**, [C]; **b**, [CIA], **c**, [FM], **d**, [FS], **e**, [F2], **f**, [F3], **g**, [VP]. Components [O_2_] and [MP] are shown in Fig. 5

Simulation plots showing the concentrations of each iron-containing component of the C_9_ model (except for nanoparticles) is shown in Fig. 4. As expected, simulated concentrations of most components increased as the nutrient iron concentration increased. Simulated concentrations of cytosolic and vacuolar components in ΔΔ cells were higher than in WT cells, whereas the simulated concentrations of mitochondrial components *FS* and *FM* in ΔΔ cells were lower than in WT cells. Vacuolar iron is dominated by *F2* under iron-deficient conditions and by *F3* under iron-sufficient ones.

### Effect on O_2_ on mitochondrial nanoparticles

**Fig. 5:**
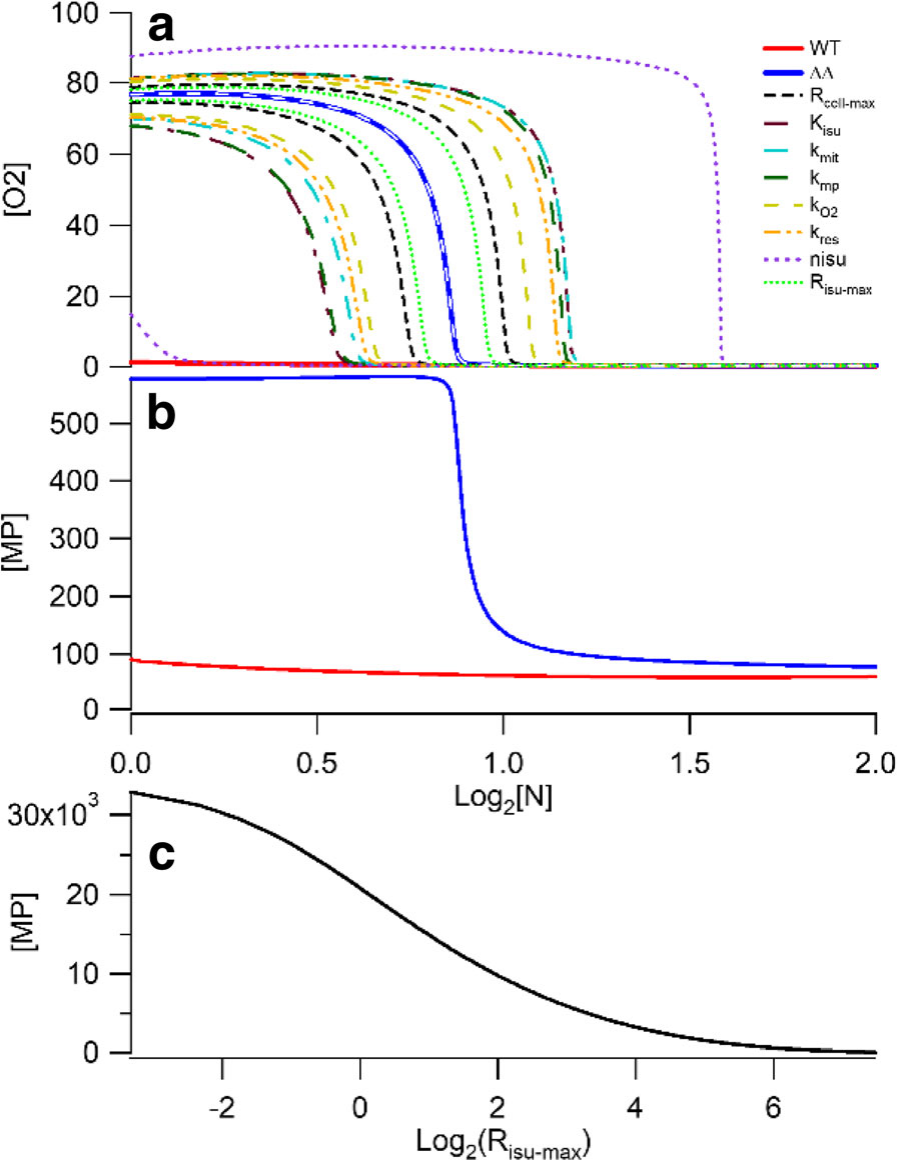
Dependence of nutrient iron on the O_2_ and nanoparticle concentrations (in μM). Optimized simulated [O_2_] (in Panel **a**) and [MP] (in Panel **b**) in mitochondria of ΔΔ (solid blue line) and WT (solid red line) cells, plotted against the nutrient iron concentration (Log_2_[N]). This is the central plot. Other plots in Panel **a**, on either side of the optimized plot, show that only certain parameters affect curve shape and position. For the other traces, the color-coded parameters shown on the side were altered ±10% of their optimized values while holding all other parameters fixed. Changing other parameters yields traces (e.g. *k*_*23*_ in the white dashed line) that had no effect on the plots and so the trace overlaid the optimal trace in the center. Panel **c** is a plot of [MP] vs. *R*_*isu-max*_, the maximum rate of *FS* formation. Low *R*_*isu-max*_ values simulate the slow rate of ISC assembly in yfh1Δ cells, while higher values reflect WT conditions

Simulations of mitochondrial O_2_ and nanoparticle concentrations are shown in Fig. 5. There is a nonlinear effect that simulates the observed behavior of ΔΔ mitochondria. Mitochondria from iron-deficient ΔΔ cells contain mostly nanoparticles and are responsible for the slow-growth defect. However, mitochondria from these cells recover when ΔΔ cells are grown in iron-sufficient medium. The plot simulates this recovery. As [N] increases, [O_2_] levels decline because increasing concentrations of respiratory complexes *FS* prevent O_2_ from diffusing into the matrix and reacting with *FM*. This allows more *FS* to be made which allows even less O_2_ into the matrix. This vicious cycle leads to the observed nonlinear behavior. The same behavior is observed for the formation of nanoparticles (Fig. 5, panel b). Other traces to either side of the best-fit [O_2_] trace represent the effect of increasing/decreasing one parameter while keeping all others fixed. Since the percentage change for each parameter was the same, the parameters that influence the shape of the plot more dramatically are located on the extremes; those that have little influence on the plot are located near the central optimized curve. A similar effect is obtained by lowering *R*_*isu*_ (Fig. 5, panel c) which simulates the effect of lowering the Yfh1 concentration in yeast mitochondria (or the frataxin concentration in human mitochondria). WT mitochondria do not exhibit this nonlinear effect because they can exclude O_2_ from the matrix at all [N] considered.

## Conclusions

### Comparison to previous model

The model developed here represents a major advance relative to our previous model [17]. Both simulate iron import and trafficking in a growing yeast cell, both include three regions (cytosol, mitochondria, and vacuoles), and both import a single nutrient iron form *N*. The major difference between the two is their level of complexity, method of optimization, and predictive power. The previous model included ~ 38 adjustable parameters (Table S2 of [17]) whereas the current C_9_ model includes only 25 (Table 2). The previous model was optimized by guessing an initial set of values and minimizing an error function. The current model was optimized using the multi-tiered approach detailed above. Perhaps the most important difference is that the current model predicts the nonlinear O_2_-dependent behavior described above while the previous model does not. Our current model was solved at different levels of complexity. We solved the simpler variants first, and discovered that the parameters obtained could be transferred to the more complex variants. This multi-tiered strategy was helpful because the parameters obtained by fitting the simpler models used more reliable data. Although the heuristics used to find optimal parameter sets do not mathematically guarantee that they globally minimize the error function, our approach yielded an acceptable solution that is compatible with known biology.

Another strategic difference in modeling approaches was that we excluded all but one Reg function in the current model. This made the current model more responsive to changing parameters and allowed better comprehension of inherent behavior.

In the end, only four parameters differed between ΔΔ and WT simulations, namely *R*_*cyt-max*_, *k*_*mit*_, *k*_*vp*_, and *K*_*α*_. *All other assigned parameter values were identical between the two genetic strains.* The ability of the model to reproduce ΔΔ and WT behavior with such few differences in terms of modeling parameters is remarkable. Moreover, we can easily rationalize why at least half of these parameters should be different. A 4.6-fold reduction of *k*_*mit*_ for ΔΔ cells makes sense because Mrs3/4, the high-affinity importers into mitochondria, are deleted in this strain. *R*_*cyt-max*_ is 2-times higher for ΔΔ cells because iron is dysregulated in these cells so expression of the Ftr1/Fet3 complex on the plasma membrane should be higher. Explaining why *K*_*α*_ should be 30-times higher in ΔΔ cells is more difficult. *K*_*α*_ is a *K*_*M*_-like parameter which reflects the sensitivity of the growth rate to changes in the nutrient iron concentration [N]. For some reason, the growth of iron-deficient ΔΔ cells is 30 times less sensitive to increases in [N] than are comparable WT cells. Perhaps this reflects difficulties in flowing sufficient iron into iron-deficient ΔΔ mitochondria to support robust respiratory cell growth. Why *k*_*vp*_ is 2-fold higher in ΔΔ cells is even more difficult to explain; it implies that the rate of vacuolar nanoparticle formation is faster in ΔΔ cells than in WT cells. The actual mechanism of vacuolar nanoparticle formation is undoubtedly more complicated than is represented in our current model. However, it is a tribute to the model that it has the ability to highlight this effect.

### Predictive power of the model

Mathematical models *might* have predictive power, but this is not guaranteed. This ability is related to how closely the assumed mechanism and assigned kinetic parameters correspond to reality. We have attempted to make our model predictive by keeping it simple and well-grounded experimentally. This was a challenge given the complexity of the process under investigation and the limited amount of relevant data available.

Our model can be used to predict the effect of O_2_ on iron metabolism in yeast cells. It predicts that the iron in mitochondria of Yfh1-deficient cells that have been grown under micro-aerobic (low O_2_) conditions should predominantly be *FM* (i.e. NHHS Fe^II^). We are currently examining a Yfh1-deficient strain of yeast, and found that NHHS Fe^II^ rather than nanoparticles are indeed observed in these cells (data not shown). Our model also predicts that O_2_ should not affect vacuolar iron (it should still be present mainly as *F3* (Fe^III^) under micro-aerobic conditions). However, this prediction is not realized by our current experiments (data not shown), highlighting a deficiency in this particular aspect of the model. We believe that this iterative approach of prediction→testing→remodeling will gradually yield major new insights in understanding iron import, trafficking, and regulation in eukaryotic cells. We are currently using this approach in our studies of the Yfh1-deficient strain.

The same strategy could be applied to model the import and trafficking of any micronutrient within any growing eukaryotic cell, from yeast to human cells. The concentration of the nutrient (or its derivatives) in whole cells and in various organelles and cytosol should be known as should exponential growth rates. Obvious candidates include other metals such as Cu, Mn, Zn, Mo, Co. The same approach could be used to examine the import and trafficking of Pt anticancer drugs into growing human cells. A better understanding of how such drugs are trafficked intracellularly might provide new insights for treating cancer.

## Additional file

Additional file 1: This file includes ODEs for the different model variants. (DOCX 61 kb)

## Footnotes

1 The abbreviations used are: CD, central doublet; CIA, cytosolic iron-sulfur assembly complex; ISC, iron-sulfur cluster; MB, Mössbauer; NHHS, nonheme high-spin; ODE, ordinary differential equation; ROS, reactive oxygen species; WT, wild-type. C_1_, C_3_, C_4_, and C_9_ refer to model variants; C_1_ includes one component called *Fe*_*Cell*_; C_3_ contains three components called *Fe*_*cyt*_, *Fe*_*mit*_, and *Fe*_*vac*_; C_4_ contains four components called *C*, *CIA*, *Fe*_*mit*_ and *Fe*_*vac*_; C_9_ contains nine components called *C*, *CIA*, *F2*, *F3*, *VP*, *FM*, *FS*, *MP*, and *O*_*2*_. Parameters *f*_*cyt*_, *f*_*mit*_, and *f*_*vac*_ are volume fractions representing the ratios V_cyt_/V_cell_, V_mit_/V_cell_, and V_vac_/V_cell_, respectively. ΔΔ is the Mrs3Δ/Mrs4Δ double-deletion strain. *α*_*cell*_ is the growth rate of the cell, and *N* is the nutrient iron species that is imported into the cell.
