# Supplementary material for: A mathematical model of iron import and trafficking in wild-type and Mrs3/4ΔΔ yeast cells
Source: BMC Syst Biol. 2019 Feb 21;13:23. doi: 10.1186/s12918-019-0702-2 (PMC6385441; doi:10.1186/s12918-019-0702-2)
Supplement: Supplementary file 1 — This file includes ODEs for the different model variants. (DOCX 61 kb) [file 12918_2019_702_MOESM1_ESM.docx]

**Additional file 1**

**ODE for the C_1_ Model:**

$$\begin{matrix} \frac{d[Fe_{cell}]}{dt}=\frac{R_{\text{cell-}\max}[N]^{sens}}{K_{N}^{sens}+[N]^{sens}}-\alpha_{cell}[Fe_{cell}] (S1) \\ \alpha_{cell}=\frac{\alpha_{\max}[N]}{K_{\alpha}+[N]} (S2) \end{matrix}$$

**ODEs for the C_3_ Model:**

$$\begin{matrix} \frac{d[Fe_{cyt}]}{dt}=\frac{R_{\text{cyt-}\max}[N]^{sens}}{K_{N}^{sens}+[N]^{sens}}-k_{mit}[C]-\frac{R_{\text{vac-}\max}[C]^{nvac}}{K_{vac}^{nvac}+[N]^{nvac}}-\alpha_{cell}[Fe_{cyt}] (S3) \\ \frac{d[Fe_{mit}]}{dt}=\frac{f_{cyt}}{f_{mit}}k_{mit}[C]-\alpha_{cell}[Fe_{mit}] (S4) \\ \frac{d[Fe_{vac}]}{dt}=\frac{f_{cyt}}{f_{vac}}\frac{R_{\text{vac-}\max}[C]^{nvac}}{K_{vac}^{nvac}+[N]^{nvac}}-\alpha_{cell}[Fe_{vac}] (S5) \end{matrix}$$

and equations (S6) and (S7) to obtain [C].

**ODEs for the C_4_ Model**

$\frac{d[C]}{dt}=\frac{k_{cyt}[N]^{sens}}{K_{N}^{sens}+[N]^{sens}}-\frac{k_{vac}[C]^{nvac}}{K_{vac}^{nvac}+[C]^{nvac}}-k_{mit}[C]-\frac{k_{cia-\max}[C]^{ncia}}{K_{cia}^{ncia}+[C]^{ncia}}-\alpha_{cell}[C] (S6)$ $\frac{d[CIA]}{dt}=\frac{k_{cia-\max}[C]^{ncia}}{K_{cia}^{ncia}+[C]^{ncia}}-\alpha_{cell}[CIA] (S7)$

and equations (S4) and (S5) to obtain [Fe_mit_] and [Fe_vac_].

**ODEs for the C_9_ Model:**

Equations (S6) and (S7) and...

$$\frac{d[F2]}{dt}=\frac{f_{cyt}}{f_{vac}}\frac{R_{vac-\max}[C]^{nvac}}{K_{vac}^{nvac}+[C]^{nvac}}-k_{23}[F2]\left(\frac{1}{1+\left(\frac{[FS]_{sp}}{[FS]}\right)^{n23}}\right)-\alpha_{cell}[F2] (S8)$$

$$\frac{d[F3]}{dt}=k_{23}[F2]\left(\frac{1}{1+\left(\frac{[FS]_{sp}}{[FS]}\right)^{n23}}\right)-k_{vp}[F3]^{nvp}-\alpha_{cell}[F3] (S9)$$

$$\frac{d[VP]}{dt}=k_{vp}[F3]^{nvp}- a_{cell}[VP] (S10)$$

$$\frac{d[FM]}{dt}=\frac{f_{cyt}}{f_{mit}}k_{mit}[C]-\frac{R_{isu-\max}[FM]^{nisu}}{K_{isu}^{nisu}+[FM]^{nisu}}-k_{mp}[FM][ O_{2}]-\alpha_{cell}[FM] (S11)$$

$$\frac{d[FS]}{dt}=\frac{R_{isu-\max}[FM]^{nisu}}{K_{isu}^{nisu}+[FM]^{nisu}}-\alpha_{cell}[FS] (S12)$$

$$\frac{d[MP]}{dt}=k_{mp}[FM][O_{2}]-\alpha_{cell}[MP] (S13)$$

$$\frac{d[O_{2}]}{dt}=k_{O2}(100-[O_{2}])-k_{mp}[FM][ O_{2}]-k_{res}[FS][O_{2}]-\alpha_{cell}[O_{2}] (S14)$$
